# The Combinational Effect of Inulin and Resveratrol on the Oxidative Stress and Inflammation Level in a Rat Model of Diabetic Nephropathy

**DOI:** 10.1016/j.cdnut.2023.102059

**Published:** 2023-12-10

**Authors:** Farideh Ghavidel, Hamed Amiri, Masoud Homayouni Tabrizi, Soodeh Alidadi, Hossein Hosseini, Amirhossein Sahebkar

**Affiliations:** 1 Department of Clinical Biochemistry, Faculty of Medicine, Mashhad University of Medical Sciences, Mashhad, Iran; 2 Department of Biology, Mashhad Branch, Islamic Azad University, Mashhad, Iran; 3 Department of Pathobiology, Faculty of Veterinary Medicine, Ferdowsi University of Mashhad, Mashhad, Iran; 4 Biotechnology Research Center, Pharmaceutical Technology Institute, Mashhad University of Medical Sciences, Mashhad, Iran; 5 Applied Biomedical Research Center, Mashhad University of Medical Sciences, Mashhad, Iran

**Keywords:** diabetic nephropathy, inulin, resveratrol, inflammation, oxidative stress

## Abstract

**Background:**

Using inulin can enhance resveratrol’s effects by improving the intestinal microbiome and the stability of resveratrol.

**Objectives:**

We aimed to investigate the effect of therapeutic intervention with combined inulin and resveratrol on kidney function in diabetic rats.

**Methods:**

Diabetic model was induced by intraperitoneal injection of streptozotocin. Afterward, rats were divided into 6 groups: control, diabetic without treatment, diabetic treated with insulin, diabetic treated with resveratrol, diabetic treated with inulin, and diabetic treated with a combination of inulin and resveratrol. After 10 wk, the creatinine, urea, insulin, urinary proteins, and inflammatory and oxidative stress markers were evaluated. Pathologic changes were examined in kidney tissues.

**Results:**

Renal dysfunction, accompanied by increased inflammation and oxidative stress, was observed. Our results showed that treatment with resveratrol and inulin had antidiabetic effects and was associated with reduced renal dysfunction, oxidative stress, and kidney inflammation. In addition, it was observed that combined treatment with inulin and resveratrol outperformed monotherapies in improving kidney function and reducing oxidative stress and inflammation.

**Conclusions:**

Treatment with resveratrol and inulin can have renoprotective effects by improving oxidative stress and inflammation in kidney tissues. Therefore, employing these 2 compounds is suggested as an inexpensive and available method for diabetic nephropathy.

## Introduction

Diabetic nephropathy (DN), one of the microvascular complications of diabetes, is the main cause of a high mortality rate in patients with diabetes [1]. It is estimated that a quarter of patients with type 1 diabetes progress to end-stage renal disease (ESRD) due to DN-related complications [2]. DN affects ∼20% to 50% of patients with type 2 diabetes [3]. DN has become the leading cause of kidney failure, with >40,000 deaths among adults worldwide in 2017 [4]. In the early stages of the disease, patients are often asymptomatic. By developing nephropathy, patients present with symptoms of fatigue, foamy urine (urinary protein excretion >3.5 g/d), and edema due to hypoalbuminemia and nephrotic syndrome [5]. So far, the exact cause of DN remains unknown, but a set of factors such as insulin resistance, genetics, hyperglycemia, and autoimmune mechanisms may be involved [[6], [7], [8]]. In addition, changes in the microbial composition of the gut can be involved in the pathogenesis of DN through the “gut-kidney axis” through alterations in the levels of short-chain fatty acids (SCFAs) in the intestine [9].

Among the causes of DN, the role of oxidative stress and inflammation are pivotal. In pathologic conditions, the overproduction of reactive oxygen species (ROS) can affect the kidney structure and function [10]. These changes ultimately lead to inflammation, fibrosis, endothelial dysfunction, and subsequent ESRD [11]. In addition, endothelial glycocalyx is the main target of ROS, which can alter glomerular filtration [12]. Renal inflammation, which involves important inflammatory pathways such as macrophages, NF-kB, and JAK/STAT, is another crucial factor [13,14]. In DN, the cycle of cytokine release, along with monocyte and macrophage recruitment, culminates in structural changes associated with inflammation. Therefore, leukocytes enter the cell, and proinflammatory cytokines such as IL-1, TNF-α, and interferon-gamma (INF-γ) can induce chemokine production, including that of IL-8, monocyte chemoattractant protein-1, chemokine (C-X-C motif) ligand-10, macrophage inflammatory protein-1, and Regulated upon Activation, Normal T Cell Expressed and Presumably Secreted (RANTES), which leads to an increase in leukocyte migration to the kidney and an inflammatory cycle is established [13,15,16]. Oxidative stress and inflammation are inseparable in physiologic and pathologic conditions. Complex interactions between oxidative stress and inflammation create a mutual feedback loop that acts as a “vicious cycle” [17]. After the activation of inflammation, immune system cells secrete cytokines and proinflammatory chemokines that stimulate ROS production and reduce antioxidant factors. Therefore, reducing oxidative stress and inflammation can be effective in improving many complications of diabetes, including nephropathy [18].

Despite recent advances, optimal control of diabetes and its complications remains a goal to be achieved. Because common treatments for diabetes can leave adverse effects, complementary or alternative treatments are recommended [19]. Herbal medicine is one of the subgroups of complementary and alternative medicine and has a long history of treating and preventing diseases, including diabetes and its complications [20].

Resveratrol or 3,5,4'-trihydroxystilbene is a natural polyphenol that different plants synthesize in response to stressful stimuli such as infection [21]. Resveratrol is found in various plants and fruits, including red grapes, blueberries, peanuts, hops, pistachios, and berries [22]. Resveratrol effects include anti-inflammatory, antioxidant, hepatoprotective, neuroprotective, cardioprotective, skin protection, antiobesity, antidiabetes, and anticancer, as shown by preclinical and clinical research [[23], [24], [25], [26], [27], [28], [29]], though conflicting findings have also been reported [[30], [31], [32]]. During the studies conducted on the antidiabetic effects of resveratrol, it has been found that this compound has antihyperglycemic effects [33]. Resveratrol appears to do so by increasing the function of the glucose transporter in the cytoplasmic membrane [34,35]. Also, resveratrol leads to strengthening adiponectin expression to increase insulin sensitivity in adipose tissue, induction of intestinal incretin hormone glucagon-like peptide-1 by the intestine, reduction of apoptosis of pancreatic β cells, and activation of Sirtuins [36]. In addition to resveratrol’s beneficial effects on diabetes, it can improve its complications, such as nephropathy [37].

But unlike the very beneficial effects of resveratrol in diabetes, commercial use of resveratrol as a drug currently faces several limitations. Polyphenols are known as xenobiotics in the human body, and their low bioavailability and rapid metabolism are considered some of the most limiting factors [38]. It has been reported that after dissolving in water, resveratrol is only stable under acidic conditions. As a result, it can be stated that the bioavailability and effects of polyphenols largely depend on their transformation by the components of the intestinal microbiota [39]. During the investigations, it has been determined that using polyphenols along with plant fibers, such as inulin can strengthen their function through the effect on the intestinal microbiome [40].

Inulin is a water-soluble storage polysaccharide that belongs to a group of indigestible carbohydrates called fructans [41]. Inulin has achieved GRAS status in the United States, meaning “generally recognized as safe,” and is widely present in ∼36,000 plant species, including chicory root, Jerusalem artichoke, yacon, asparagus, leek, onion, banana, wheat, and garlic [42]. As a dietary fiber, inulin remains undigested while passing through the digestive tract. It acts as a prebiotic in the large intestine because the beneficial intestinal microflora selectively ferments it and increases its growth. In addition, inulin is easily fermented by gut bacteria and produces large amounts of SCFAs, indicating the potential impact of inulin on the composition of the human gut microbiome [43].

It has been observed that inulin can lower glucose levels and improve diabetes in animal models [44]. The analysis of the study by Wang et al. [45] confirmed that 4 main glycemic indexes (fasting blood sugar, HbA1C, fasting insulin, and HOMA-IR) were significantly reduced by inulin supplementation in prediabetic and diabetic populations. Additionally, the study by Dehghan et al. [46] revealed that inulin supplementation modulates inflammation and metabolic endotoxemia in women with type 2 diabetes mellitus. Inulin mainly exerts its effects by producing SCFAs, leading to an increase in total antioxidant capacity (TAC) and superoxide dismutase (SOD) and a decrease in malondialdehyde (MDA) [47]. However, an exact mechanism for reducing oxidative stress has not been stated. Inulin can also reduce inflammation by reducing IL-6, TNF-α, plasma LPS, and CRP concentrations [48].

As we know, intestinal microbiome changes are associated with kidney disease. In ESRD, intestinal flora bacteria change significantly [49]. Because inulin has few side effects and is effective in modulating the intestinal microbiome, it can be used as a prebiotic to reduce the complications of DN [50]. In the investigations conducted on the effect of the combined use of inulin and polyphenols, studies have shown that these 2 compounds interact with each other to modulate the intestinal microbiome and create favorable conditions for the microbiome in the intestine by increasing beneficial intestinal bacteria and SCFAs. Due to the low absorption of resveratrol in the digestive system, its simultaneous use with inulin can effectively increase its bioavailability with reduced pH of the intestinal environment, including its decomposition, absorption, transfer, and release [51]. Therefore, in this study, we have investigated the individual and combined effects of resveratrol and inulin and compared them with individual effects in nephropathy animal model rats.

### Animals

In this study, 48 male Wistar rats (body weight: 300–350 gm, age: 7–8 wk) obtained from the Pasteur Institute of Iran were used. They were kept at a temperature of 22 ± 2 °C and a light/dark cycle of 12 hrs in the Mashhad University of Medical Sciences animal house. Resveratrol and Inulin were both purchased from Sigma-Aldrich. The SOD enzyme activity, MDA, and TAC assay kits were from Teb Pazhouhan Razi. The GeneAll Hybrid-R RNA purification kit was purchased from GeneAll Biotechnology. RevertAid First Strand cDNA Synthesis Kit was purchased from Thermo Fisher Scientific.

All experiments were conducted according to institutional ethics and in compliance with international animal testing regulations under the code of ethics IR.MUMS.MEDICAL.REC.1400.789.

### Experiment design

Diabetes was induced by injecting a single dose of 65 mg/kg streptozotocin, intraperitoneally, which was obtained from Sigma-Aldrich (St. Louis). Three days after the induction of diabetes, plasma glucose was measured using a glucometer. Diabetic rats included animals with fasting glucose >250 mg/dL. The studied population consisted of 6 groups of 8 rats. The grouping of rats was as follows: group 1: control group consuming a standard cow diet (SCD); group 2: diabetic rats receiving streptozotocin (65 mg/kg), group 3: diabetic rats receiving streptozotocin (65 mg/kg) and then daily administration of neutral protamine Hagedorn (NPH) insulin (3 IU/d) for 10 wk; group 4: diabetic rats receiving streptozotocin (65 mg/kg) and daily resveratrol (20 mg/kg) by gavage for 10 wk; group 5: diabetic rats receiving streptozotocin (65 mg/kg) and daily inulin (100 mg/kg) by gavage for 10 wk; and group 6: diabetic rats receiving streptozotocin (65 mg/kg) and a daily combination of inulin (100 mg/kg) and resveratrol (20 mg/kg) by gavage for 10 wk. After 10 wk, all animals were sacrificed after ether anesthesia to remove kidney tissues. The kidneys were stored at −80 °C for further measurements. Blood was also collected from the heart for biochemical tests.

### Measuring body weight and amount of water and food consumption

Food and water consumption was measured daily during the study period, and body weight was measured at the end of the study. The rats were weighed using an electronic scale. The food consumed was determined by measuring the difference between the preweighed food and the remaining food weight every 24 h. Water consumption was measured by recording the water remaining in the corresponding bottle.

### Urinary protein measurements

A day before dissection, rats were kept individually and separately in metabolic cages for 24 h, and urine was collected for protein excretion examination.

### Histopathology

For histopathologic examination, kidney tissue samples were first weighed and then fixed in 10% formalin buffer for 12 h. In the next step, the samples were dehydrated using alcohol and molded in paraffin. Then, tissue sections were prepared and stained using hematoxylin and eosin staining. Tissue sections were 5-μm thick.

### Investigating the level of oxidative stress

The SOD enzyme activity, MDA, and TAC levels were investigated using commercial kits (Teb Pazhouhan Razi).

### RT-PCR

A TRIzol reagent (Invitrogen) was used to extract total RNA from the kidney tissues following the manufacturer’s recommendations. Then, cDNA was synthesized using a RevertAid First Strand cDNA Synthesis Kit (Thermo Fisher Scientific) from an aliquot of whole RNA.

Relative expression of target genes was determined by qRT-PCR using SYBR Green Master Mix (Thermo Fisher Scientific). The results were normalized to glyceraldehyde 3-phosphate dehydrogenase expression level, and 2^−ΔCT^ was used to compare the relative expression of target genes between groups. The primer sequences are listed in Table 1.

**Table 1:** Details of the primer pairs used in RT-qPCR

Gene	Sequence
Fw – GAPDH	AGTGCCAGCCTCGTCTCATA
Rv – GAPDH	TGAACTTGCCGTGGGTAGAG
Fw – TNF-α	ACACACGAGACGCTGAAGTA
Rv – TNF-α	TTCCGGGATCCAGTGAGTTC
Fw – IL-1β	GACTTCACCATGGAACCCGT
Rv – IL-1β	GGAGACTGCCCATTCTCGAC
Fw – IL-6	CCTTCTTGGGACTGATGTTGTT
Rv – IL-6	TATACTGGTCTGTTGTGGGTGG
Fw – NRF2	TGGATCTGTCAGCTACTCCCA
Rv – NRF2	ATCCAGGGCAAGCGACTCAT
Fw – HO-1	ATGACACCAAGGACCAGAGC
Rv – HO-1	GTGTAAGGACCCATCGGAGA

Abbreviation: HO-1, heme oxygenase-1

### Statistical analyses

All statistical analyses were performed by GraphPad Prism 9.4.0 software using one-way analysis of variance and then Tukey’s multiple comparisons to compare different groups. The normal distribution of all data in this study was evaluated using the Kolmogorov-Smirnov test. Data were represented as mean ± SD, and *P* value of <0.05 was considered statistically significant.

### The effect of resveratrol in combination with inulin on blood glucose and insulin level in diabetic rats

**FIGURE 1:**
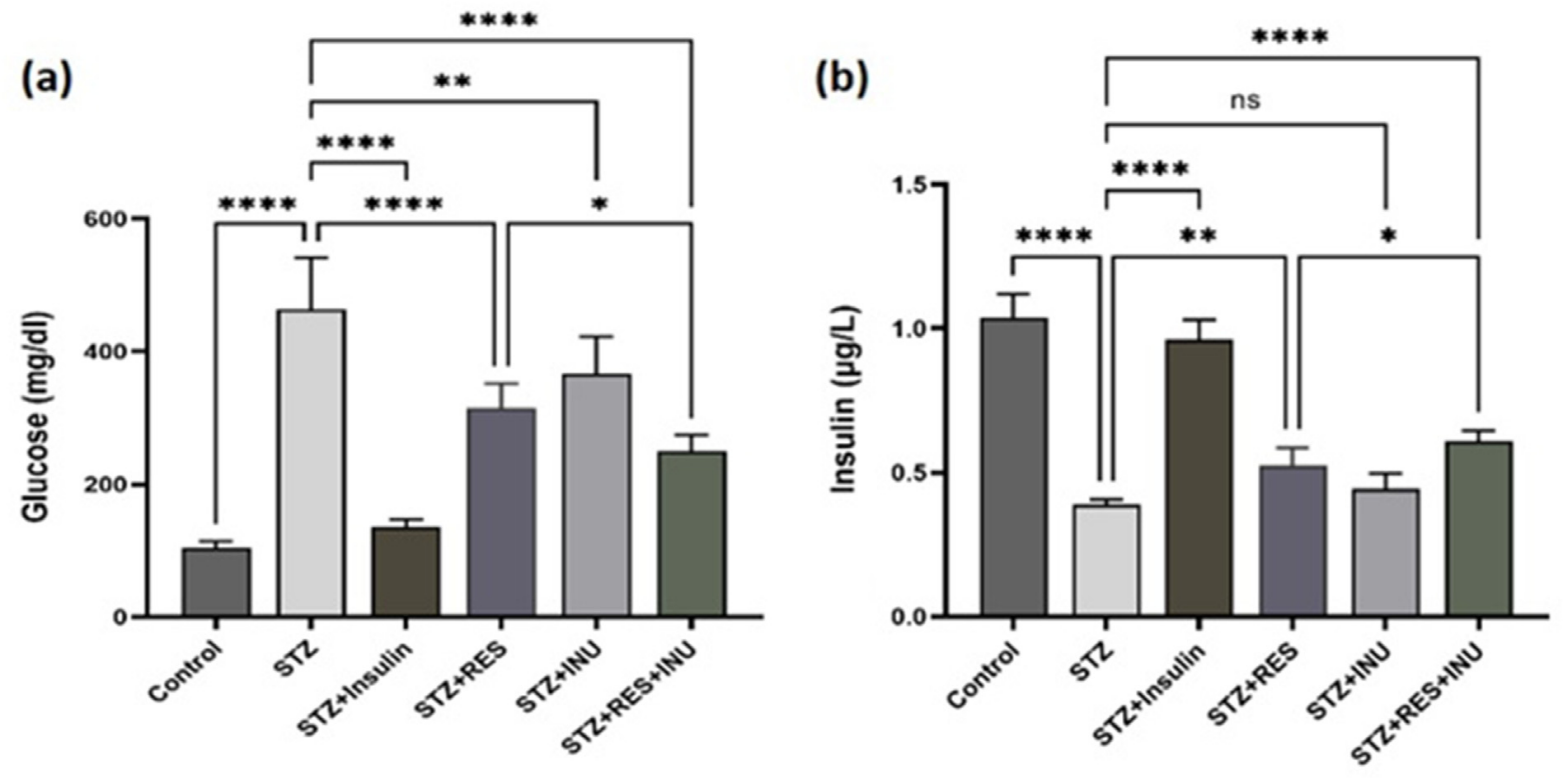
The effect of resveratrol treatment in combination with inulin on (A) blood glucose and (B) insulin levels in diabetic rats. After 10 wk of streptozotocin treatment, the rats’ blood glucose was measured using a glucometer, and plasma insulin levels were measured in the blood plasma of the rats. Data are expressed as mean ± SD. ∗∗∗∗*P* < 0.0001, ∗∗∗ *P* < 0.001, ∗∗ *P* < 0.01, ∗ *P* < 0.05.

In the present study, to create a DN model in rats, streptozotocin was injected intraperitoneally at a dose of 65 mg/kg. As expected, blood glucose significantly increased when compared with that of the treatment group. It is noteworthy that combined treatment in the resveratrol+inulin group (25.46 ± 291.8 mg/dL) showed a significant decrease when compared with the resveratrol+streptozotocin group, which indicates the stronger effect of the combined treatment (*P* < 0.0001) (Figure 1A). Furthermore, the streptozotocin administration leads to the destruction of pancreatic β cells and causes diabetes. In this study, it was observed that in the group of untreated diabetic rats, plasma insulin (0.39 ± 0.015 μg/L) was significantly decreased when compared with the control group (1.036 ± 0.82 μg/L). In this study, resveratrol somewhat improved the plasma insulin level, but inulin could not increase it. In addition, in the combination treatment group, insulin was associated with a significant increase, which was more effective than the single resveratrol treatment (*P* < 0.05) (Figure 1B).

### The effect of resveratrol in combination with inulin on the amount of food and water intake, kidney and body weight in diabetic rats

**FIGURE 2:**
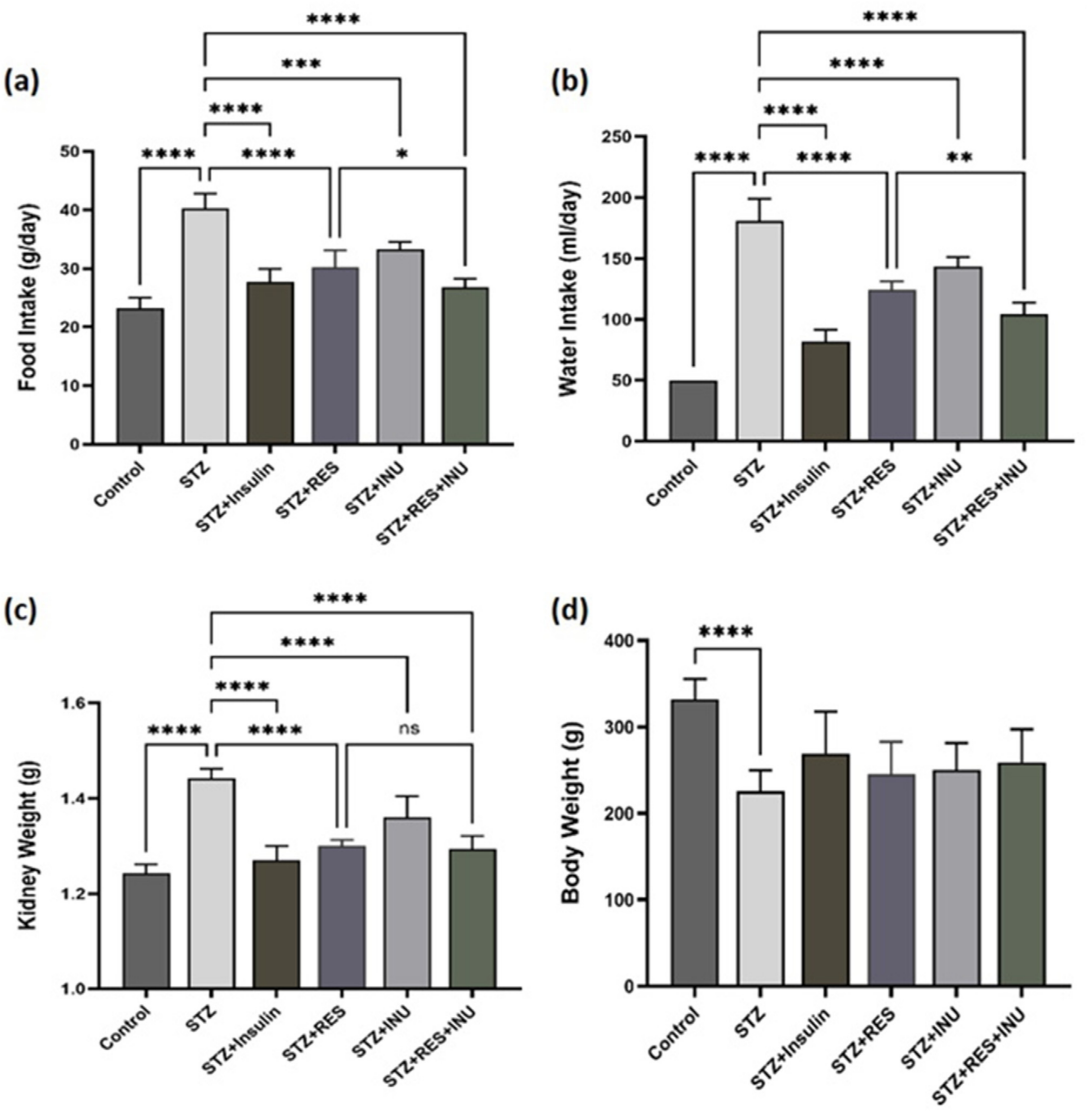
The effect of resveratrol treatment in combination with inulin on (A) food and (B) water intake, (C) kidney, and (D) body weight in diabetic rats. After initiating the treatment for the rats, the daily amount of food and water consumption of the rats was measured during the study, and the body and kidney weight of each rat was measured separately at the end. Data are expressed as mean ± SD. ∗∗∗∗*P* < 0.0001, ∗∗∗ *P* < 0.001, ∗∗ *P* < 0.01, ∗ *P* < 0.05.

An increase in food consumption is expected due to the effects of diabetes on the hunger centers. Also, because of hyperglycemia and frequent urination, an increase in water consumption is predictable. This study showed that resveratrol, inulin, and their combination significantly reduced food and water consumption in diabetic rats (*P* < 0.0001). It was also observed that reducing food and water consumption in the combined treatment group had a better effect when compared with the resveratrol or inulin treatment group alone (Figure 2A and B). In addition, the development of type 1 diabetes in rats can lead to metabolic disorders and subsequent weight loss. In this study, to investigate the effects of inulin, resveratrol, and their combination on body weight, rats were weighed at the end of 10 wk of treatment. As expected, the results showed that the body weight of the rats in the untreated diabetic group significantly decreased when compared with the controls. However, no significant improvement was observed in any of the treatment groups. In addition, due to nephropathy, the kidney tissues had undergone fibrosis, which increased its weight. Therefore, kidney weight was measured separately in each rat. The results showed a significant increase in kidney weight in the diabetic group when compared with the control group, which was significantly reversed in all treatment groups. However, in this case, the inulin-resveratrol combination did not have a significant effect compared with resveratrol alone (Figure 2C and D).

### The effect of resveratrol in combination with inulin on biochemical parameters related to kidney function in diabetic rats

#### Effect on plasma urea and creatinine levels and 24-h urinary protein excretion

**FIGURE 3:**
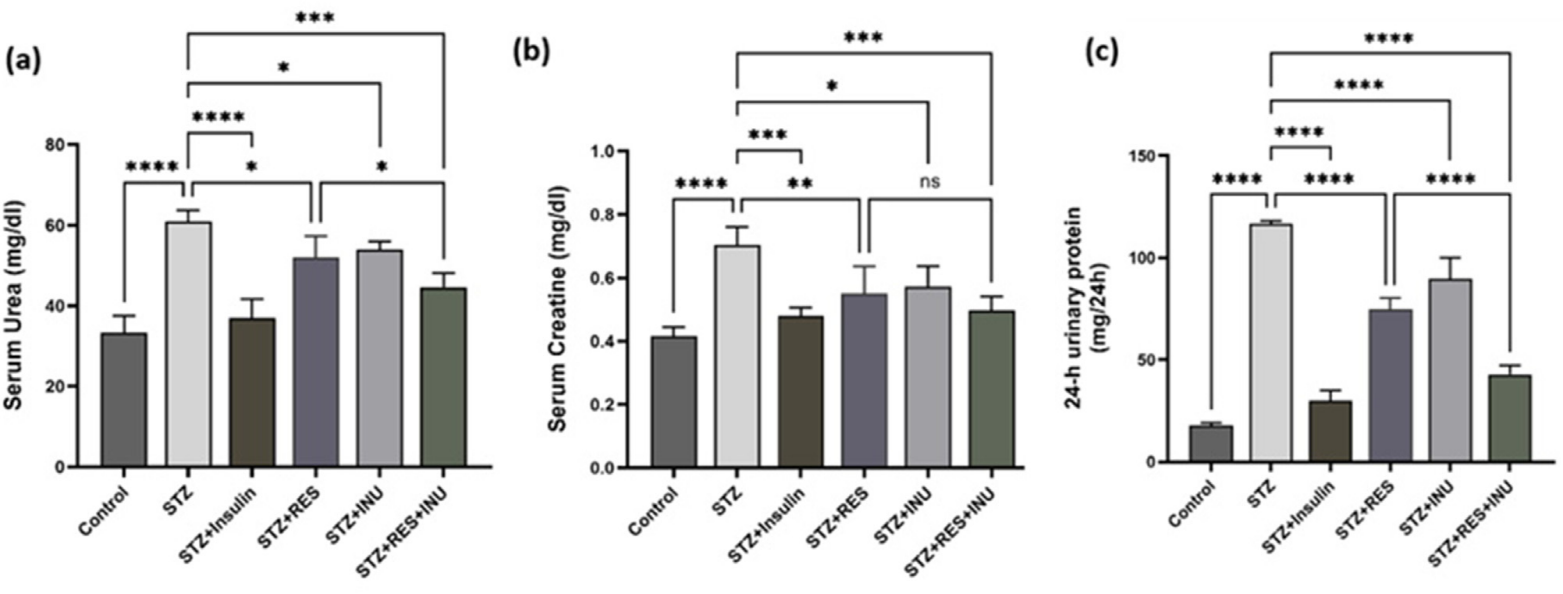
The effect of resveratrol treatment in combination with inulin on (A) urea, (B) creatinine, and (C) 24-h excretion of urinary protein levels in diabetic rats. At the end of the study, blood was collected from the hearts of the rats, and the serum sample was separated. Then it was used to measure urea and creatinine. Furthermore, 24-h urinary protein was collected using a metabolic cage. Data are expressed as mean ± SD. ∗∗∗∗*P* < 0.0001, ∗∗∗ *P* < 0.001, ∗∗ *P* < 0.01, ∗ *P* < 0.05.

Plasma urea and creatinine are important biochemical parameters that increase kidney damage. The average of these parameters has increased significantly due to the creation of a diabetic model and kidney damage in untreated diabetic rats. Urea and creatinine in the resveratrol and inulin treatment group significantly decreased when compared with the untreated diabetic group (*P* < 0.05). Also, regarding urea, a significant difference was observed between the resveratrol and inulin combined treatment group and the resveratrol alone treatment group, but the difference in creatinine was not significant when compared with the resveratrol treatment group. Our findings indicate the improvement of renal biochemical parameters in diabetic conditions by combined treatment with inulin and resveratrol (Figure 3A and B). Also, due to extensive kidney damage in diabetes, the excretion of urinary proteins increases in rats. In this study, 24-h urine samples were collected by metabolic cages to check the rate of excretion of urinary proteins. The results showed that the excretion of urinary proteins in the 24-h urine sample of untreated diabetic rats (116.7 ± 0.88 mg/24 h) compared with the control group (17.67±0.88 mg/24 h) had a significant increase, which indicates kidney damage. A significant decrease in the excretion of urinary proteins is observed in the resveratrol and inulin treatment group and their combination, which was more effective in the combined treatment group when compared with the individual treatment groups (Figure 3C).

#### Histopathologic results

**FIGURE 4:**
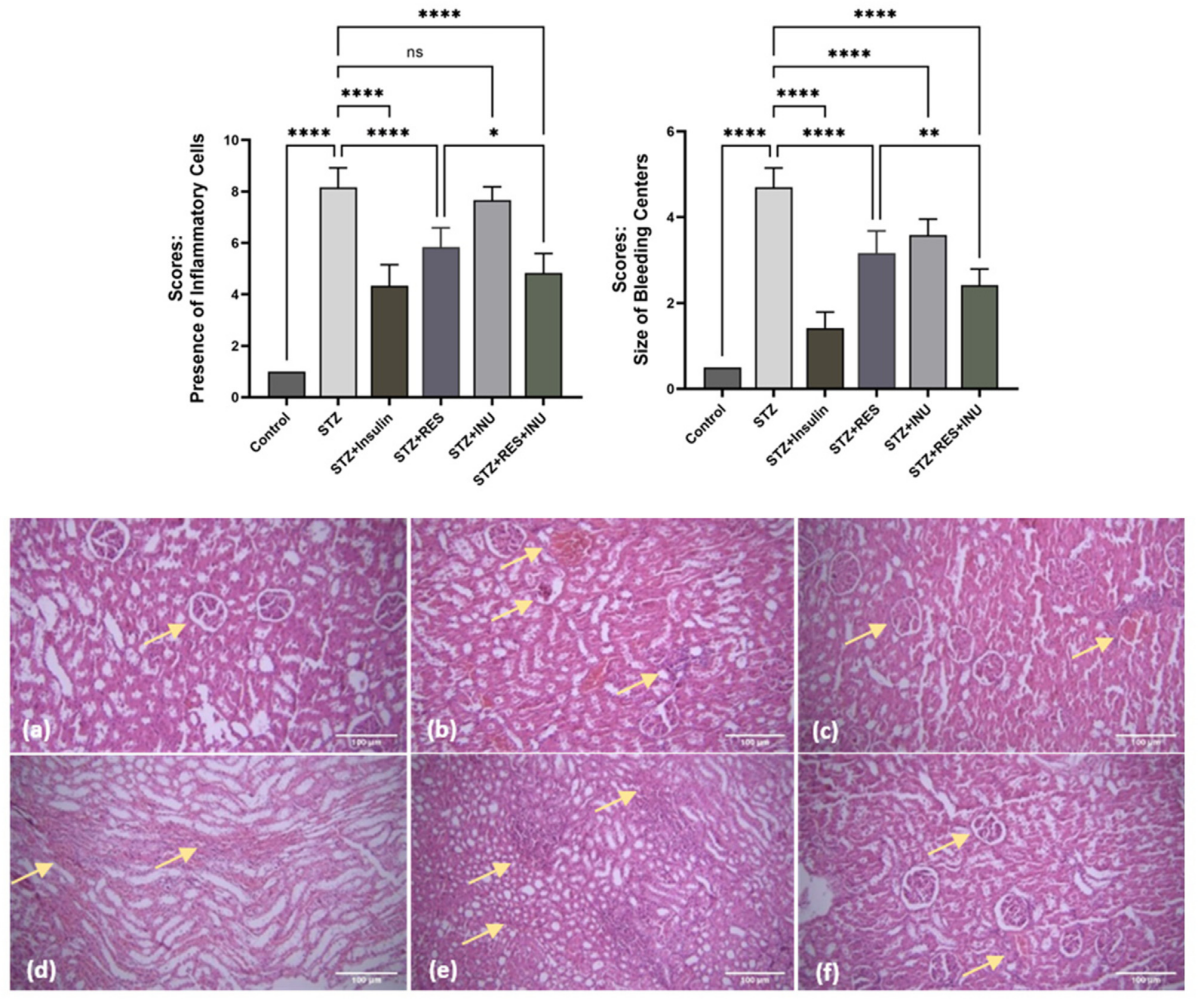
Histologic changes in the kidneys of diabetic rats along with scores (A): the normal structure of the kidney’s central part with sections of renal tubules in group 1. Hematoxylin and eosin staining. 100× magnification. (B): Presence of bleeding foci, degenerative and necrotic changes, infiltration of inflammatory cells, and glomerular atrophy in untreated diabetic kidney tissue of group 2. Hematoxylin and eosin staining. 100× magnification. (C): The presence of a small focus of bleeding and inflammatory reaction in the cortical part of the kidney tissue of group 3. Pay attention to the normal size of kidney glomeruli. Hematoxylin and eosin staining. 100× magnification. (D): Bleeding and mild inflammation in the central part of the kidney tissue of group 4. Hematoxylin and eosin staining. 100× magnification. (E): The presence of inflammatory foci in the kidney tissue of group 5 receiving inulin. Hematoxylin and eosin staining. 100× magnification. (F): The normal size of glomeruli and the reduction of cell swelling in the cortical part of the kidney tissue. Hematoxylin and eosin staining in kidney tissue of group 6. 100× magnification.

In examining the changes and histopathologic lesions of kidney tissues related to different study groups, the following observations were recorded: group 1, control: the microscopic structure of kidney tissue was observed as normal (Figure 4A). Group 2: diabetic rats without treatment: changes observed in this group include degeneration and necrosis of the lining cells of the kidney tubes, hyperemia, relatively severe bleeding in some areas of the cortex and center of the kidney, atrophy, infiltration of inflammatory cells, as well as foci of accumulation of inflammatory cells in the place of tubules, atrophy of glomeruli in the kidney capsule (Figure 4B). Group 3: diabetic rats with insulin injection (positive control): histopathologic lesions observed in this group were significantly reduced when compared with group 2, and tissue order and integrity were improved (Figure 4C). Group 4: diabetic rats receiving resveratrol: the lesions observed in tissue sections related to this group included mild bleeding, atrophy of glomeruli, and degenerative and necrotic changes in kidney tubule lining cells. Also, the intensity of cellular swelling of the lining cells of kidney tubules was reduced (Figure 4D). Group 5: diabetic rats receiving inulin: the lesions observed in tissue sections related to this group included mild bleeding, atrophy of glomeruli, and degenerative and necrotic changes in kidney tubule lining cells. Also, the intensity of cellular swelling of the lining cells of kidney tubules was reduced (Figure 4E). Group 6: diabetic rats receiving combined treatment: In the histopathologic examination of the kidney tissue related to this group, hyperemia, mild bleeding, improvement of changes in degeneration and necrosis of tubule lining cells, mild atrophy of glomeruli, and mild lymphocytic nephritis were observed (Figure 4F)

#### The effect of resveratrol in combination with inulin on the expression of Nrf2 signaling genes and oxidative stress indices in kidney tissue of diabetic rats

**FIGURE 5:**
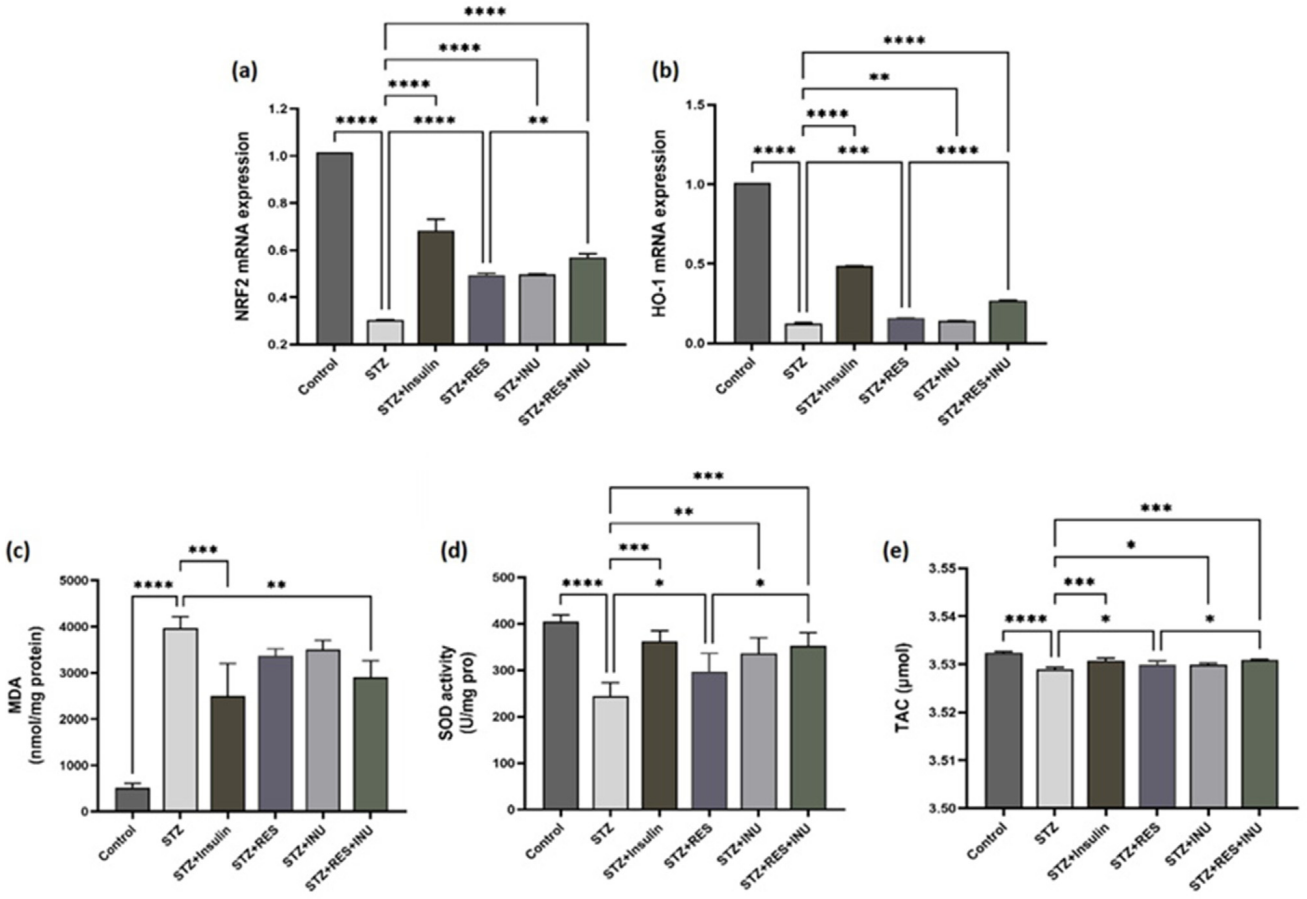
The effect of resveratrol treatment in combination with inulin on (A) Nrf2 and (B) HO-1 expressions and (C) MDA, (D) SOD, and (E) TAC in kidney tissue of diabetic rats. At the end of the study, the kidney tissues of the rats were isolated, and the mRNA expression levels of Nrf2 and HO-1 genes in the kidneys was investigated by RT-PCR method. Besides, oxidative stress factors, including SOD, MDA, and TAC, were examined in the kidney tissue of rats. Data are expressed as mean ± SD. ∗∗∗∗*P* < 0.0001, ∗∗∗ *P* < 0.001, ∗∗ *P* < 0.01, ∗ *P* < 0.05. HO-1, heme oxygenase-1; MDA, malondialdehyde; STZ, streptozotocin; RES, resveratrol; INU, inulin, Nrf2, nuclear factor erythroid 2–related factor 2; SOD, superoxide dismutase; TAC, total antioxidant capacity.

The Nrf2 signaling pathway is essential in controlling the gene expression of antioxidant factors. The expression of antioxidant factors, including heme oxygenase-1 (HO-1), increases when this pathway is activated. In this study, we examined gene expression of Nrf2 factors and its downstream antioxidant enzyme HO-1 in kidney tissue. It was observed that these 2 factors together in the tissues of all diabetic rats without treatment had a significant decrease, which was improved by the treatment with inulin and resveratrol and led to an increase in the expression of these genes and antioxidant activity. In addition, the improvement of the expression of Nrf2 and HO-1 in the combined treatment with inulin and resveratrol is also observed when compared with the treatment alone (Figure 5A and B). Among the important factors in the evaluation of oxidative stress indicators, we can mention SOD, MDA, and TAC. For this purpose, in this study, SOD, MDA, and TAC were measured in the kidney tissues of rats. As a marker of lipid peroxidation, MDA was significantly increased in the group of diabetic rats without treatment. Only in the combined treatment group was a significant decrease, and resveratrol and inulin alone did not lead to significant improvement. In addition, SOD levels decreased in the untreated diabetic rats when compared with the control group. SOD activity was significantly improved by resveratrol and inulin treatment alone, which is more significant in the combined treatment group. In addition, TAC in the group of rats without treatment had a significant decrease, which resveratrol and inulin treatment and combined treatment led to a significant improvement (Figure 5C–E).

#### The effect of resveratrol and inulin combination on the expression of TNF-α, IL-1β, and IL-6 genes

**FIGURE 6:**
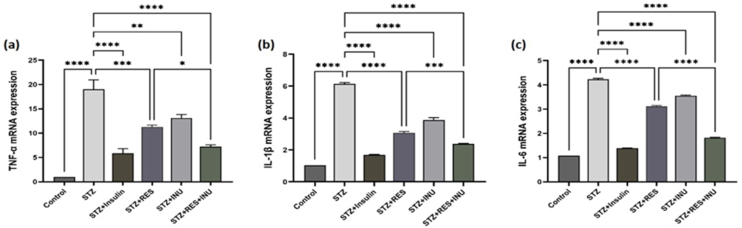
The effect of resveratrol treatment in combination with inulin on (a) TNF-α, (b) IL-1β, and (c) IL-6 expression in kidney tissue of diabetic rats. At the end of the study, the kidney tissue specimens of the rats were isolated, and the mRNA expression level of TNF-α, IL-1β, and IL-6 genes in the kidney was evaluated by RT-PCR. Data are expressed as mean ± SD. ∗∗∗∗*P* < 0.0001, ∗∗∗ *P* < 0.001, ∗∗ *P* < 0.01, ∗ *P* < 0.05.

Evidence has confirmed that diabetes is associated with a chronic inflammatory condition. A part of chronic inflammation is due to hyperglycemia through changes in the characteristics of cytokines and chemokines. To understand the effect of resveratrol and inulin compounds on inflammatory responses, we measured the mRNA expression of some key cytokines. The results showed that the expression of IL-6, TNF-α, and IL-1β genes in the kidney tissue of untreated diabetic rats increased significantly when compared with the control group. In the treatment group of resveratrol and inulin alone, the expression of these inflammatory cytokines in the kidney tissues significantly decreased. Data analysis shows that the resveratrol and inulin combination was more effective in diabetic rats (Figure 6A–6C).

DN is one of the main causes of death in patients with diabetes [52]. Its first clinical phenotypes include microalbuminuria, and an early detection of this complication can help the patient prolong the development of ESRD [53]. Although strong evidence suggests the benefits of blood glucose and blood pressure control in preventing DN, the disease risk is not reduced. The results obtained from the treatment of DN have not changed significantly, and this complication is still a major concern, especially for endocrinologists and nephrologists [2]. Recently, natural herbal compounds have become attractive therapeutic agents in diabetes because they have fewer side effects than currently used diabetes drugs [54].

Resveratrol and inulin have antidiabetic, anti-inflammatory, and antioxidant effects. However, the low bioavailability of resveratrol and its rapid metabolism is a big obstacle to its effects on the human body [55]. Besides, increased dosage and repeated dosing of resveratrol do not significantly alter its bioavailability [56]. Considering that the intestine and liver are the main sites of resveratrol metabolism, the colon’s bacterial metabolism effectively improves its bioavailability [56,57]. Recent studies show that dietary fibers play a role in modulating gut microbiota, and these microorganisms convert polyphenols into active metabolites with high bioavailability. Therefore, changes in the intestinal microbiota can affect the activity of polyphenols [57]. Thus, in this study, we investigated the effect of resveratrol, inulin, and their combination in the DN rat model.

This investigation revealed that combinational resveratrol and inulin treatment had healing effects on kidney-related biochemical factors, oxidative stress, and inflammation. We observed that the fasting blood glucose of diabetic rats treated with streptozotocin increased significantly. Single-dose injection with a high concentration of streptozotocin in rats leads to type 1 diabetes and subsequent hyperglycemia. However, there are several methods to induce different types of diabetes. Among these methods, adding a high-fat diet or nicotinamide along with streptozotocin can lead to type 2 diabetes. However, in this study, we were able to create diabetic nephropathy by inducing type 1 diabetes by injecting a single dose of streptozotocin [58].

The production of ROS in β cells is one of the cytotoxic mechanisms that streptozotocin can lead to in rats following increased blood glucose levels [59]. In addition, insulin deficiency also occurs due to the destruction of β cells. The resulting hyperglycemia contributes to the onset of various complications of diabetes, including kidney damage [60]. High blood glucose levels lead to abnormal homeostasis in the blood flow and vascular permeability in the glomerulus [61]. Although it is said that in the early stages, the increase in glomerular permeability is reversible, the lesions become irreversible under the continuous stimulating effect of hyperglycemia. In addition, other pathways involved in DN include increased ROS activation, increased AGE receptors that are effective for the expression of TGF-β and cytokines, which causes renal fibrosis, Protein kinase C (PKC) activation that leads to hyperplasia of glomerular basement membrane [62].

Our study observed that resveratrol and inulin, alone or in combination, effectively reduce the adverse renal effects of diabetes. The body and kidney weight measurements of diabetic animals receiving resveratrol or inulin were significantly higher when compared with diabetic animals receiving no treatment. These data show that the treatment is effective on body metabolism and prevents weight loss along with kidney tissue hypertrophy.

Various studies have shown the relationship between hyperglycemia with body or kidney weight. The weight loss of diabetic animals can be due to the harmful effects of streptozotocin administration, which causes DNA alkylation, hyperglycemia, necrotic lesions, and hypoinsulinemia [63]. Also, increased kidney weight is because of glomerular hypertrophy, which is effective in the progression of glomerular damage [64]. Although the exact mechanism of renal hypertrophy is unknown, evidence suggests that local changes are due to the production of growth factors or overexpression of TGF-β, particularly in cells of the proximal convoluted tubule and glomeruli [65]. In our study, treatment with resveratrol, inulin, and their combination inhibited body weight loss, but this result was nonsignificant. Moreover, the weight of the kidneys in the treatment groups had decreased when compared with the streptozotocin group without treatment, which can indicate improvement of renal hypertrophy caused by diabetes. This significant difference was not observed in the combined treatment group when compared with the group receiving resveratrol alone. Consistent with our study, Sharma et al. [66] observed that resveratrol effectively reduced body weight in diabetic rats. In another study by the same author, it was observed that resveratrol reversed and reduced the effects of streptozotocin-induced kidney weight gain [67].

Furthermore, in diabetic rats, the amount of food and water consumption was increased in the streptozotocin group, which shows improvement in the resveratrol, inulin, and combined treatment group. Additionally, these 2 factors show significant improvement in the combined treatment group when compared with the group receiving resveratrol alone. Resveratrol also effectively improves plasma insulin levels, which is more visible when combined with inulin. Mechanistically, resveratrol can increase insulin production by protecting β cells with its antioxidant activity. Resveratrol is also said to reduce the autoimmune destruction of these cells, which leads to an increase in the number of islets of Langerhans [68,69]. In addition, we found that resveratrol reduced blood urea and creatinine, as well as urinary protein excretion. These effects show a better improvement in combination with inulin, except for the creatinine factor, which indicates the increased effects of resveratrol on the kidney when used with inulin. In line with our study, a clinical trial has shown that resveratrol can effectively be an adjunct to angiotensin receptor blockers to reduce urinary albumin excretion in patients with DN [70].

As mentioned, the combined effect of polyphenols and inulin can result from changes in the composition of the gut microbiome and can also be done through the “gut-kidney axis.” Dysbiosis created in the gut produces and accumulates endotoxins and uremic toxins that leak through the damaged gut barrier into the systemic circulation, causing inflammation and nephrotoxicity [71,72]. On the other hand, the dysbiosis of the gut microbiome leads to a decrease in SCFAs in the intestine. The production of SCFAs under normal conditions stabilizes blood glucose levels and has protective effects on kidney cells; therefore, the reduction of SCFA in diabetes leads to kidney damage [73]. That is why improving the intestinal environment can effectively improve kidney diseases. A study has been conducted regarding the effect of inulin on treating type 1 diabetes. The production of SCFAs by inulin leads to an increase in IL-22, which accentuates the antidiabetic effects of inulin. Moreover, it has been determined that inulin is associated with reducing blood glucose, improving kidney hypertrophy, improving blood urea, reducing hyperphagia and polydipsia, reducing systemic inflammatory indices, and maintaining the composition of the intestinal microbiome. In addition, this study revealed that inulin normalized the function of β cells and improved insulin sensitivity. Thus, inulin can potentially prevent and treat type 1 diabetes [74].

One of the important factors in the development of DN is the increased production of ROS. In our study, resveratrol and inulin alone or in combination significantly improved oxidative stress indices, including MDA, SOD, and TAC. Regarding MDA, a significant change was observed only in the combination group, which can be said that the effects of the combinational resveratrol and inulin treatment can help improve the performance of resveratrol. So far, several mechanisms, including inhibiting free radicals, increasing the activity of antioxidant enzymes, inhibiting NF-κB, and activating SIRT1, have been described for the antioxidant effects of resveratrol [75]. Furthermore, inulin can diminish oxidative stress by reducing lipid peroxidation and gene regulation of different antioxidant enzymes in different tissues [76]. Bejar et al. [77] reported that inulin improved blood glucose, insulin resistance, and oxidative stress by inhibiting the α-glucosidase enzyme. In the study by Morshedi et al. [78], there was a significant increase in TAC activity and serum SOD and GPx and a decrease in serum MDA concentration after inulin consumption in diabetic rats. In this study, we concluded that resveratrol, inulin, and their combination treatment could be effective by activating the NRF2/KEAP1 pathway and increasing the production of enzymatic and non-enzymatic antioxidants.

Nrf2 is a transcription factor that regulates the expression of antioxidant genes and protects the cell against oxidative damage. The antioxidant effects associated with this pathway are associated with the activation of genes containing antioxidant response elements (ARE). KEAP1 is a regulatory protein that controls the activity of Nrf2. Without external stimulation, Nrf2 is in the cytoplasm, binds to KEAP1, and inactivates. When ROS accumulate, conformational changes in KEAP1 cause it to dissociate from Nrf2 and translocate to the nucleus. Then, musculoaponeurotic fibrosarcoma protein (Maf) forms a heterodimer with Nrf2 and finally binds to ARE to increase the expression of downstream phase II antioxidant genes and produce antioxidant enzymes [79]. This event causes the expression of Nrf2/ARE-dependent antioxidant genes such as HO-1 [80].

Resveratrol and inulin have been effective in improving inflammatory factors. These 2 compounds, whether alone or in combination, reduced the levels of inflammatory factors such as TNF-α, IL-1β, and IL-6 in diabetic rats, which can indicate the anti-inflammatory properties of these 2 drugs. Many studies have reported that resveratrol modulates the inflammatory response through different signaling pathways, such as the arachidonic acid pathway, inhibiting cyclo-oxygenase and hydroperoxides, inhibiting NF-κB activation, MAPK signaling pathways and activator protein-1 [75]. Anti-inflammatory properties have also been documented for inulin. Yasuda et al. [81] reported that the consumption of inulin can reduce the expression of genes related to inflammation, including TNF-α, in young pigs. In another study by Lecerf et al. [82], inulin supplementation for 4 wk significantly reduced the expression of inflammatory cytokines such as IL-1β or TNF-α. Additionally, the expression of anti-inflammatory cytokines such as IL-13 and IL-10 in the blood increased [82]. Similar to many studies, our study showed that the use of resveratrol and inulin was effective in reducing the levels of inflammatory cytokines. The reduction of the levels of these cytokines was more effective when the combination of resveratrol and inulin was used, which shows the synergistic effects of these 2 compounds together.

This study found that resveratrol, combined with inulin, could exert protective effects on the kidney, including reducing oxidative stress and inflammation. These effects can be mainly attributed to the improvement of resveratrol bioavailability through inulin’s effect on the intestinal environment. In addition, combined resveratrol and inulin treatment in diabetic rats was more effective than treatment with either of the compounds alone. Therefore, the use of resveratrol and inulin, in combination with standard treatment, might be effective in improving some of the complications of DN. Further investigations are required to confirm the clinical efficacy of this intervention, as well as the molecular mechanisms mediating the observed protective effects of resveratrol and inulin.

## Author contributions

The authors’ responsibilities were as follows –

## Conflict of interest

The authors report no conflicts of interest.

## Funding

Supported by grants awarded by the Mashhad University of Medical Sciences (4001377) to H.H. All authors have read and approved the manuscript.

## Data availability

Data described in the manuscript will be available from the first and corresponding authors on a reasonable request.

## Appendix A Supplementary data

Multimedia component1 Multimedia component1
