# Supplementary material for: The Combinational Effect of Inulin and Resveratrol on the Oxidative Stress and Inflammation Level in a Rat Model of Diabetic Nephropathy
Source: Curr Dev Nutr. 2023 Dec 10;8(1):102059. doi: 10.1016/j.cdnut.2023.102059 (PMC10826146; doi:10.1016/j.cdnut.2023.102059)

Supplementary figure 1. Hematoxylin and eosin staining of kidney tissues of rats in various groups (100x and 400x).


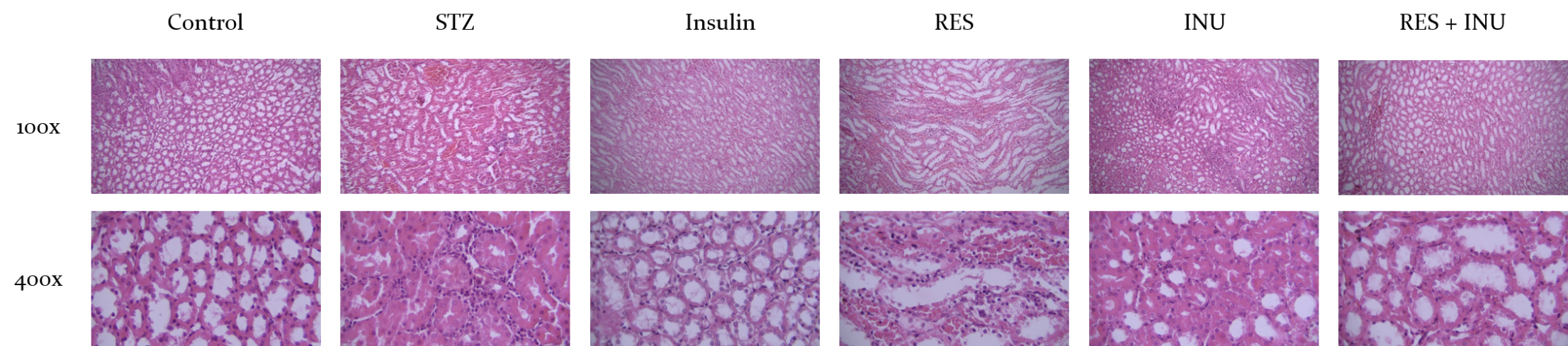

Supplement: Multimedia component1 [file mmc1.docx]
